# Migration status, physical limitations and associated self-rated health: a study of older Indian adults

**DOI:** 10.1186/s12877-023-04002-0

**Published:** 2023-05-22

**Authors:** Bittu Mandal, Kalandi Charan Pradhan, Parimala Mohanty, T. Muhammad

**Affiliations:** 1 School of Humanities and Social Sciences, Indian Institute of Technology Indore, Khandwa Road, Simrol, 453552 Indore India; 2 Institute of Medical Sciences & Sum Hospital, Siksha “O” Anusandhan, Bhubaneswar, 751030 Odisha India; 3 Department of Family & Generations, International Institute for Population Sciences, Mumbai, 400088 Maharashtra India

**Keywords:** Migrant status, Functional/mobility impairments, Self-rated health, Older adults, India

## Abstract

**Background:**

Migrant status with mobility impairment becomes a double burden for health and wellbeing of older adults. This study examined the independent relationships and multitude effects between migrant status, functional and mobility impairments and poor self-rated health (SRH) among older Indian adults.

**Methods:**

This study utilised nationally representative Longitudinal Ageing Study in India wave-1 (LASI) data, including a sample of 30,736 individuals aged 60 years and above. The main explanatory variables were migrant status, difficulty in activities of daily living (ADL), difficulty in instrumental activities of daily living (IADL) and mobility impairments; and the outcome variable was poor-SRH. Multivariable logistic regression and stratified analyses were used to fulfil the study objectives.

**Results:**

Overall, about 23% of older adults reported poor-SRH. Reporting poor-SRH was more prevalent (28.03%) among recent migrants (less than ten years). The prevalence of reporting poor-SRH was significantly higher among older adults who had mobility impairment (28.65%), difficulty in ADL or IADL (40.82% & 32.57%). Migrant older adults (regardless of duration) who had mobility impairment had significantly greater odds of reporting poor-SRH compared with non-migrant older adults who did not have mobility impairment. Similarly, older respondents who had problems in ADL and IADL with migration status had higher odds of reporting poor-SRH than their non-migrant counterparts with no such problems.

**Conclusions:**

The study revealed the vulnerability of migrant older adults with functional and mobility disability, as well as those with limited socioeconomic resources and suffering from multimorbidity on rating their perceived health. The findings can be utilised to target outreach programmes and provision of services for migrating older individuals with mobility impairments and enhance their perceived health and ensure active ageing.

## Background

The World Health Organization (WHO) estimates that the share of older individuals will be more than double from 12 to 22% by 2050, and the low- and middle-income nations will host 80% of all older adults globally [1]. The proportion of persons aged 60 years and older in India will increase from 9% of the total population in 2015 to about 19% in 2050 [2]. On the other hand, India has witnessed a staggering increase in the number of internal migrants in the last few decades. In 2001, India accounted for around 309 million internal migrants, which rose to as many as 450 million in 2011, and it was expected around 600 million people in India migrated internally in 2021 [3]. Internal migration has a substantial impact on the social, economic, and overall health and well-being of older people [4].

Though migration results in social and economic mobility, many a time, this comes with adverse impacts on health. There is a growing body of literature in India that explored several types of health impairments of the people with migrant status. A large number of studies investigated the health status of migrant populations relating to a particular disease, i.e., HIV [5–7], malaria [8, 9], obesity and diabetes [10, 11]and cardiovascular disease [12, 13]. Much of the literature is based on adults, relating to specific subpopulations such as slum dwellers [14], construction workers [15], and workers engaged in transportation [16, 17]. It is not surprising that a multitude of migrants’ health studies shows that migrants tend to report worse self-assessed health compared to non-migrants [18, 19]However, health inequalities between migrants and non-migrants do not follow a unitary pattern, some studies found that migrants tend to report better self-reported health than their native counterparts [20–22]. Since migration functions in conjunction with other health factors, poor socioeconomic status has frequently been shown as the driving force of health adversities [23, 24]. Place of residence was also associated with migrants’ health [23], and many studies demonstrate that gender appears as an additional factor intersecting with migration in determining adverse health consequences [19, 20].

However, only limited research used self-rated health (SRH) to measure overall health and health disparity between migrant and non-migrant population. On the other hand, SRH is a widely used indicator of general health and is a consistent predictor of morbidity and mortality [23, 25, 26]. It is based on a single survey question, which proved to be a valid and reliable measure to assess the overall health of the population [27–29], recommended by the US Centres for Disease Control, the World Health Organization, and the European Commission for use in health monitoring [30–32]. It is also reliable in the Indian context [24]. Most of the older people,whether they have internally migrated or stayed in their original location, are outside the social safety net. As a result, they confront economic and health insecurity and inequality, posing a strain to an already saturated societal system [33]. Moreover, in the old age, mobility is a key pillar of self-sufficiency and a “hallmark of ageing”. Mobility limitation is common among older adults, and it worsens with age [34, 35]. Mobility limitation, on the other hand, has been associated with a lower quality of life and poor psycho-social health [36, 37]. Mobility impairment in particular, relates to a loss of independence, a lower quality of life, and a higher risk of death in older persons [38, 39].

The functional component of maintaining an active and independent daily life involves synthesizing various subjective evaluations related to mental health, ability to maintain social and family relations, and perceived overall health [40, 41].Negative self-perceived health has an amplifying effect and is significantly associated with functional limitation. Functional limitation is linked with a decreased capacity for self-care and social role fulfilment, which may lead to feelings of dependence and undermine autonomy, ultimately contributing to negative self-perceived health [42, 43]. Likewise, research has established that physical functioning measures, such as activities of daily living (ADL) or instrumental activities of daily living (IADL), are correlated with feelings of well-being. Any inability to carry out these activities has been found to cause a decline in self-rated health among older adults [43, 44].

Research on functional/mobility impairment and self-rated health is rare and poorly understood in developing countries and is yet to be well appreciated [45]. For older adults, migrant status with functional/mobility impairment becomes a double burden for their health and wellbeing. Furthermore, studies demonstrating the effects of older adults’ migration and functional/mobility impairments on their health status have been centred on the global north; however, whether their findings are consistent in the global south, especially in the Indian setting, is unknown. By filling this knowledge gap, this cross-sectional study aimed to investigate the associations of migration status and functional/mobility impairments with poor-SRH among older adults using a large country-representative survey data in India. We further examined the multitude effects of migrant status and functional/mobility impairments on poor-SRH among older adults by doing the stratified analysis.

## Methods

### Data

The study is based on the data from the Longitudinal Ageing Study in India (LASI) wave 1 (2017–18), a nationwide longitudinal large-scale survey of ageing and health. In the first wave of the LASI, 72,250 people aged 45 and above, as well as their spouses of any age, were interviewed across all Indian states and union territories (excluding Sikkim), with 31,464 older adults (aged 60 and up) respondents. The LASI survey’s primary aim was to investigate the health and social and economic well-being of India’s old age population. LASI delivers anonymized scientific data on a population health, social, mental, and economic well-being that is valid, reliable, and continuous. To arrive at the final units of observation, the LASI used a multistage stratified area probability cluster sampling design. In rural areas, LASI used a three-stage sample design, whereas in urban areas, it used a four-stage sample design. The national report of LASI, wave 1, 2017–18, India, contains detailed information on the sampling framework and sample size selection.

### Study sample

**Fig. 1:**
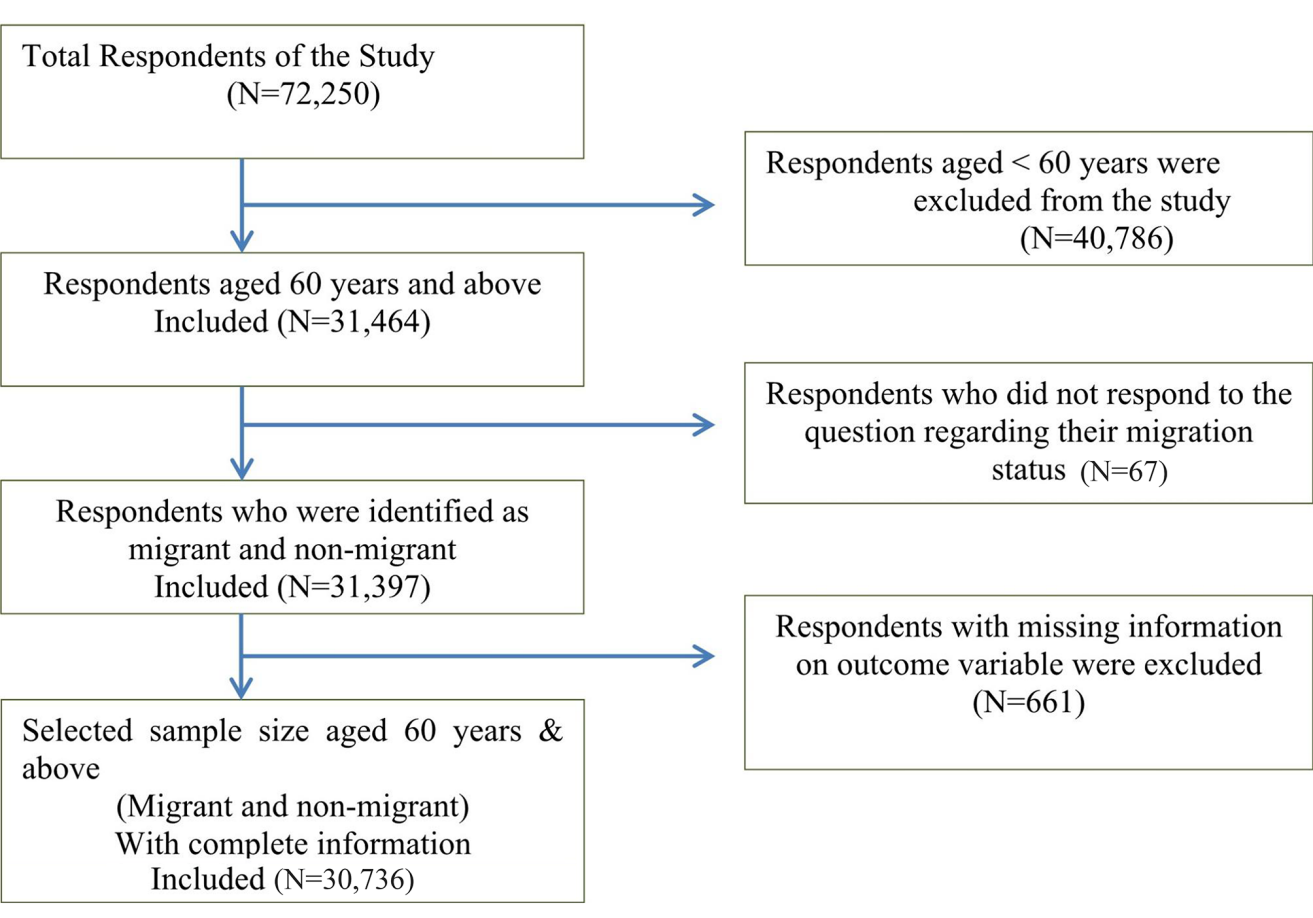
Selection criteria of the sample study

The LASI wave 1 provides data on a total sample of 72,250 people aged 45 and up and their spouses, regardless of age, with no missing values in age reporting. The participants in our study were older individuals, aged 60 and up, who either migrated or stayed at the same place since birth. As a result, the sample of those under the age of 60 was eliminated (n = 40,786). Those who responded “Since Birth” to the survey question “How many years have you been living (continuously) in this area” were classified as non-migrant, and others as Migrant. In this line of argument, 67 Respondents were excluded from the study sample as they did not respond to the above survey question regarding their Migration status. The dependent variable was self-reported health. Missing values were dropped in case of respondents who did not reply to the self-reported health questions, as a result, 661 respondents were removed from the study sample. Thus, the effective sample size was 30,736 older individuals age 60 years or older. The complete sample selection criteria for the study are shown in Fig. 1.

### Variable description

#### Outcome variable

The outcome variable was poor self-rated health (SRH). We utilize an individual’s SRH status as a measure of his or her overall health. Although SRH is a subjective measure, it offers a holistic assessment of a person’s health. It has been demonstrated that it can predict mortality and disability [23, 25]. Many previous studies have also used SRH status to measure the health of individuals [46–48]. The following question was used in the LASI to measure participants “Overall, how is your health in general?“ which had responses of “very good,“ “good,“ “fair,“ “poor,“ and “very poor.“ For the present study, the responses were categorized into two groups and were recorded as “1” for the responses of very poor and poor, representing a poor-SRH and “0” for rest of the responses of excellent, very good and good representing a good-SRH.

### Explanatory variables

#### Main explanatory variable

Migration status: We considered migration status as one of our main independent variables. Migration status was determined by the answer on the question in the survey “How many years have you been living (continuously) in this area?” Respondents who stated that they had lived in the place since birth were defined as non-migrants and others as migrants. To get a clear picture of the effect of migration on the health outcomes of older individuals, we classified the migration status following the duration of residence in the destination place. We categorised the migrants into two broad categories: (a) migrants who were living at the destination place for less than ten years, and (b) migrants who were living in the destination place for ten or more years. Thus, the migration status of the respondents finally considered for this study was: non-migrant (living in the area since birth), migrant for ten or more years and migrant for less than ten years.

Mobility Impairment: Mobility refers to the physical ability to move, which is often necessary for participating in meaningful social, cultural, and physical activities. Mobility is also important for accessing public facilities and can be significant for self-care. To assess the mobility impairment among older adults, we considered nine self-reported items, available in the LASI survey, including:(a) Walking 100 yards; (b) Sitting for 2 h or more; (c) Getting up from a chair after sitting for long period; (d) Climbing one flight of stairs without resting; (e) Stooping, kneeling or crouching; (f) Reaching or extending arms above shoulder level (either arm); (g) Pulling or pushing large objects; (h) Lifting or carrying weights over 5 kilos, like a heavy bag of groceries; (i) Picking up a coin from a table. The response for the questions was available as ‘no’ and ‘yes’. Scale was summed up and was recoded into a dichotomous measure as ‘0’did not have any mobility difficulty and ‘1’otherwise.

Difficulty in ADL refers to at least one difficulty in ADLs which include regular self-care tasks. Assessing a person’s ability or inability to perform ADLs is a means of measuring their functional status, particularly for individuals with disabilities or older adults. To assess ADL limitations, respondents were asked if they were having any of the following limitations and they anticipated any of the following limitations to continue longer than three months: difficulty with dressing, walking across the room, bathing, eating, getting in or out of bed, or using the toilet (including getting up and down). The respondents who had no difficulty in performing ADLs were categorised as ‘no’ (code 0) and otherwise categorised as ‘yes’ (code 1).

Difficulty in IADL was assessed using six questions asked to the respondents if they were having any difficulties that were expected to last for at least 3 months, such as (a) shopping for groceries, (b) preparing a hot meal, (c) making a telephone call, (d) doing work around the house or garden, (e) taking medications, managing money like paying bills and keeping track of expenses, and (f) getting around or finding an address in unfamiliar places. Older adults who reported no difficulty in performing IADL were assigned to the ‘no’ category (code 0), and those who reported any difficulty were assigned to the ‘yes’ category (code 1).“

Based on extensive literature review, the following independent variables were included to carry out this study.

1. Age was grouped into 60–69 years, 70–79 years and 80 + years.
2. Sex was categorised as male and female.
3. Education was recoded as No education/Primary, secondary and Higher.
4. Marital status was recorded as Married, widowed and others. The latter included those who were separated, deserted, and never married.
5. Living arrangement was recoded as living alone, living with spouse and living with others.
6. Working status was recoded as currently working and not working.
7. Social participation was measured through the question “Are you a member of any of the organizations, religious groups, clubs, or societies? Responses were coded as ‘yes’ as 0 and ‘no’ as 1.
8. Physical activity was accessed by the question ‘how often you take part in sports or vigorous activities, such as running or jogging, swimming, going to a health centre or gym.’ Frequency of doing such activities were accessed through five-point scale, where hardly ever or never coded as ‘No’ = 1 and others as ‘Yes’= 0 for this study.
9. The variable yoga/meditation was accessed from the survey question “How often do you engage in any of the following activities like yoga, meditation, asana, pranayama or similar?” and categorised as ‘no’=1 (those who reported hardly ever or never did yoga, meditation) and others as ‘yes’=0.
10. Multimorbidity refers to the coexistence of two or more chronic health conditions in a single individual. chronic diseases were assessed using the question “has any health professional ever diagnosed you with the following chronic conditions or diseases?”. Responses were available as yes and no. Hypertension, diabetes, cancer or malignant tumour, any chronic lung disease, any chronic lung disease, chronic heart diseases, stroke, any bone/joint disease, any neurological/psychiatric disease, and high cholesterol were the nine chronic health conditions which were included for the present study. Further, multi-morbidity status was categorised as ‘no’ (absence or single chronic disease), and ‘yes’ (two or more chronic disease).
11. Sleep problem was categorised as ‘Yes’ and ‘No’ assessed by the survey question, during the past 1 month, how often do you have trouble falling asleep; would you say- Never, rarely (1–2 nights per week), occasionally (3–4 nights per week), or frequently (5 or more nights per week)?” Respondents who answered ‘Never’ were recoded as ‘0’ and others as ‘1’.
12. Pain was assessed by asking respondents the question “Are you often troubled with pain” and was categorised as ‘No’=0 (Did not report pain) and others as ‘Yes’ =1.
13. The monthly per capita consumption expenditure (MPCE) quintile was determined using household consumption data, and the information linked to household-level consumption of food and non-food items was used. The reference periods for food expenditure were seven days and for non-food expenditure were 30days and 365 days. These expenditures have been standardized to the 30-day reference period. The variable was then categorised into five into five quintiles i.e., from lowest to highest.
14. Religion was coded as Hindu, Muslim, Christian and others.
15. Caste was recoded as Scheduled Castes/Scheduled Tribes (SC/ST), Other Backward Class (OBC) and others.
16. Place of residence was categorised as rural and urban.

### Statistical analysis

Descriptive statistics and bivariate analysis were used in this study to evaluate the prevalence of subjective health among the older adult respondents in the country based on socio-economic status and other characteristics. The significance level of the bivariate association was determined using the Chi-square test. In addition, binary logistic regression analysis [49] was used to look at the association between migration status and mobility impairment with poor-SRH in older adults in India.

The equation of the logistic regression is as follows:

$$\begin{array}{l}Logit\left( y \right) = \ln \frac{p}{{1 - p}}\\= \alpha + {\beta _1}{X_1} + {\beta _2}{X_2} + {\beta _3}{X_3} \ldots .{\beta _k}{X_k}\end{array}$$

The regression coefficients in this example were β1, β2… … …β_k_ and they showed the relative effect of explanatory variables and socio-demographic and health behavioural factors on the dependent variable is the residual .Unadjusted and adjusted regression models were deployed to determine the association between explanatory and outcome variables. Furthermore a set of stratification analyses between key explanatory variables and poor-SRH were also carried out after adjusting for the selected covariates to assess the multitude effects between main variables on the outcome of interest. The results were presented in the form of odds ratio (OR) and with a 95% Confidence Interval (CI). Statistical analysis was performed in stata 15 software [50].

## Results

Table 1 shows the demographic and socioeconomic characteristics of the respondents stratified by migration status in India. Five out of every ten older persons, either migrants or not, were between the ages of 60 and 69. Additionally, 39.91%, 27.03% and 59.23% of non-migrant respondents had primary or no formal education, were widowed and were currently not working, respectively. Further, among migrant respondents, 41.17% had primary education or illiterate, 42.92% were widowed, and 75.70% were currently not working. Among the respondents, 61.99% and 73.30% of the non-migrants and migrants were not physically active, respectively. Migrants (26.23%) were more multimorbid than non-migrants (20.78%). Furthermore, 16.27% and 16.71% of the non-migrant and migrant respondents belonged to the lowest strata of wealth, respectively. The majority (more than 80%) of older adults followed Hinduism, and more than one fourth of the migrant and non-migrant respondents belonged to the SC/ST caste.

**Table 1:** Socio-demographic profile of respondents stratified by migration status, India, LASI (2017–2018)

Background Characteristics	Non-migrant		Migrant		Total	
	Sample	%	Sample	%	Sample	%
Age						
Young-old (60–69)	8169	57.9	10,574	60.65	18,743	59.45
Old-old (70–79)	3981	30.31	4877	29.48	8858	29.84
Oldest-old (80+)	1426	11.79	1709	9.87	3135	10.71
Sex						
Male	9669	76.1	5104	25.18	14,773	47.33
Female	3907	23.9	12,056	74.82	15,963	52.67
Education						
No education/Primary	2688	39.91	3048	41.17	5736	40.53
Secondary	2999	42.76	3007	40.12	6006	41.46
Higher	1132	17.33	1455	18.71	2587	18.01
Marital Status						
Currently Married	9587	70.31	10,019	55.35	19,606	61.86
Widowed	3575	27.03	6764	42.92	10,339	36
Others	414	2.66	377	1.73	791	2.14
Living Arrangement						
Living with spouse	9498	69.96	9875	57.55	19,373	63.03
Living with others	3466	25.53	6304	36.74	9770	31.79
Living alone	612	4.51	981	5.72	1,593	5.18
Working Status						
Working	5321	40.77	3920	24.3	9241	31.46
Not Working	8255	59.23	13,240	75.7	21,495	68.54
Social Participation						
Yes	959	5.03	1145	4.58	2104	4.78
No	12,472	94.97	15,801	95.42	28,273	95.22
Physical Activity						
Rigorous	3063	23.28	2435	14.76	5498	18.47
Moderate	2020	14.73	1963	11.94	3983	13.15
Never	8417	61.99	12,663	73.3	21,080	68.38
Yoga meditation						
Daily	1422	10.1	1969	9.23	3391	9.61
Often	528	4.08	767	4.46	1295	4.29
never	11,547	85.82	14,314	86.31	25,861	86.1
Multi Morbidity						
No	10,714	79.22	12,407	73.77	23,121	76.14
Yes	2853	20.78	4743	26.23	7596	23.86
Sleep Problem						
No	8118	61.28	9087	51.94	17,205	56.01
Yes	5458	38.72	8073	48.06	13,531	43.99
Pain						
No	8308	63.53	9990	57.92	18,298	60.36
Yes	5259	36.47	7157	42.08	12,416	39.64
MPCE Quintile						
Lowest	2925	22.41	3379	21.27	6304	21.77
Lower	2857	22.4	3458	21.07	6315	21.65
Middle	2721	20.23	3555	21.09	6276	20.71
Higher	2650	18.69	3395	19.87	6045	19.35
Highest	2423	16.27	3373	16.71	5796	16.52
Religion						
Hindu	9628	82.36	12,911	82.91	22,539	82.67
Muslim	1764	11.94	1868	9.84	3632	10.75
Others	2184	5.7	2381	7.25	4565	6.57
Caste						
SC/ST	4889	27.59	5149	26.57	10,038	27.01
OBC	5172	46.87	6467	43.92	11,639	45.21
Others	3515	25.54	5544	29.51	9059	27.78
Residence						
Urban	3500	22.15	7001	34.67	10,501	29.22
Rural	10,076	77.85	10,159	65.33	20,235	70.78

SC/ST Schedule caste/ Schedule tribe, OBC Other backward caste, MPCEMonthly per capita consumption expenditure

**Fig. 2:**
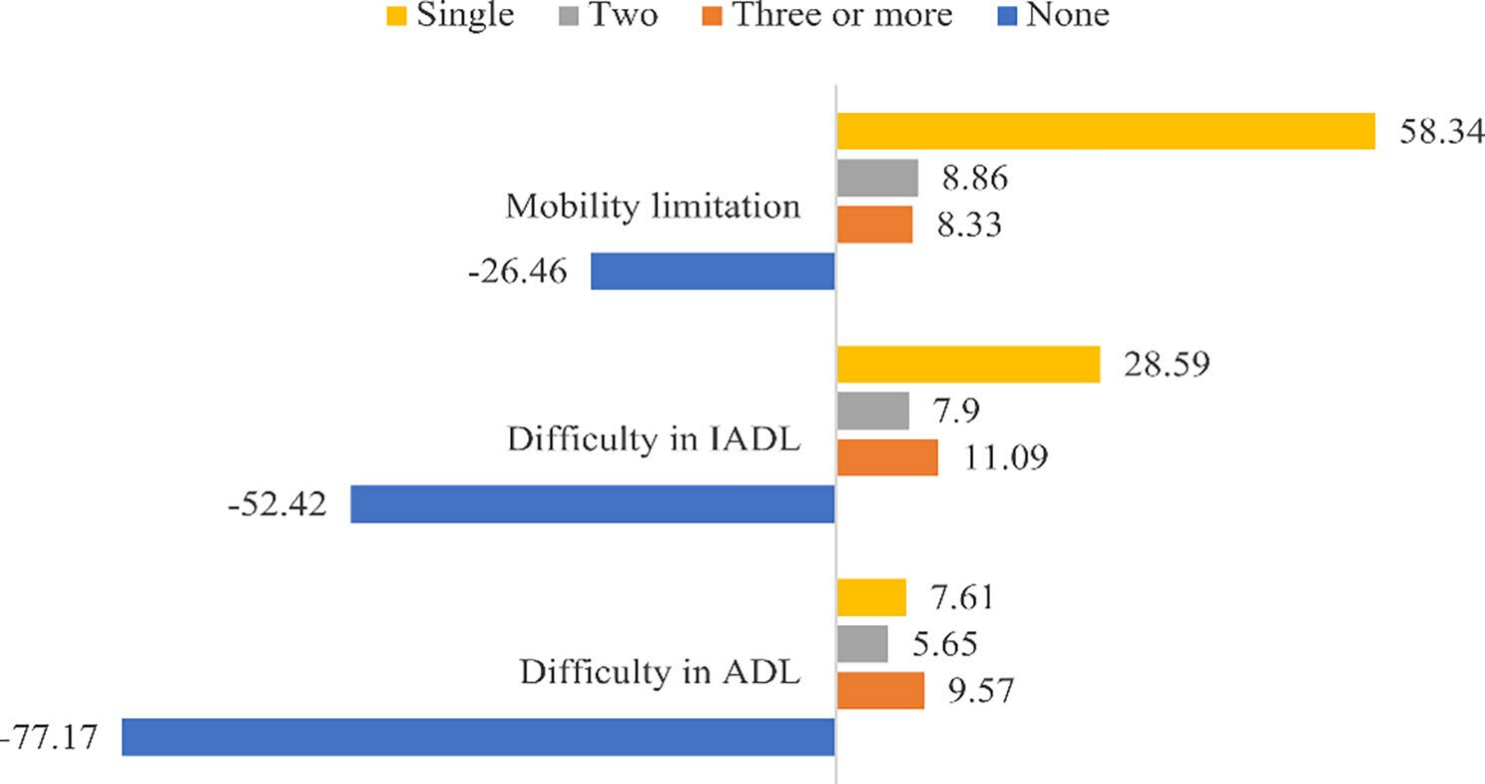
Percentage of older adults who had mobility limitation, difficulty in IADL and ADL in India

Figure 2 presents the percentage distribution of older people by their status on mobility and functional abilities. A proportion of 58.34% of older adults reported at least one mobility impairment whereas, 28.59% reported an IADL difficulty and 7.61% reported a ADL difficulty in this study.

Table 2 demonstrates the prevalence of reporting poor SRH among older adults by background characteristics. The overall prevalence of poor-SRH was 23% among older adults (n = 7093). The prevalence of reporting poor-SRH was significantly higher among the recent migrants (less than ten years) and migrants for ten years or more compared to their non-migrant peers (28.03% & 25.14% vs. 22.75%). Around 28% of older adults who had mobility impairment reported poor-SRH compared to 11% older adults who did not have mobility impairment. A higher percentage of respondents (41% and 33%) who had difficulties in ADL or IADL reported poor-SRH than their counterparts (19% and 17%) who were free from these difficulties.

**Table 2:** Unadjusted prevalence of poor-SRH by socio-economic and demographic background among old age population, India, LASI (2017–2018)

Background Factors	Poor-SRH (%)	Chi-square, p value
Migration Status		< 0.001
Non-migrant	22.75	
Migrant (10 years or more)	25.14	
Migrant (less than 10 years)	28.03	
Mobility impairment		< 0.001
No	10.56	
Yes	28.65	
ADL difficulty		< 0.001
No	19.29	
Yes	40.82	
IADL difficulty		< 0.001
No	16.67	
Yes	32.57	
Age		< 0.001
Young-old (60–69)	19.14	
Old-old (70–79)	26.91	
Oldest-old (80+)	35.76	
Sex		< 0.001
Male	20.88	
Female	25.11	
Education		< 0.001
No education/ Primary	24.66	
Secondary	20.68	
Higher	15.11	
Marital Status		< 0.001
Currently Married	20.59	
Widowed	27.23	
Others	30.59	
Living Arrangement		< 0.001
Living with spouse	23.07	
Living with others	28.03	
Living alone	38.84	
Working Status		< 0.001
Working	13.99	
Not working	26.98	
Social Participation		< 0.001
Yes	17.49	
No	23.5	
Physical Activity		< 0.001
Rigorous	12.31	
Moderate	15.44	
Never	27.32	
Yoga Meditation		< 0.001
Frequent	17.52	
Often	17.14	
Never	24.1	
Multimorbidity		< 0.001
No	18.1	
Yes	38.19	
Sleep Problem		< 0.001
No	17.43	
Yes	30.26	
Pain		< 0.001
No	16.52	
Yes	32.76	
MPCE Quintile		< 0.001
Lowest	24.16	
Lower	22.99	
Middle	21.14	
Higher	23.87	
Highest	23.26	
Religion		< 0.001
Hindu	23.49	
Muslim	25.74	
Others	18.93	
Caste		< 0.001
SC/ST	20.59	
Other backward class	25.76	
Others	22.39	
Place of Residence		0.47
Urban	22.84	
Rural	23.2	

SC/ST: Schedule caste/ Schedule tribe, OBC: Other backward class, MPCE:Monthly per capita consumption expenditure

Table 3 presents the results obtained from the logistic regression analysis of the socio-economic and lifestyle factors of poor-SRH among older adults. The odds of reporting poor-SRH were significantly higher among older adults who were recent migrants (for less than ten years) [AOR: 1.25; CI: 1.10–1.50], or migrants with a longer duration (ten years or more) [AOR: 1.08, CI: 1.01–1.15] in comparison to their non-migrant peers. The odds of reporting poor-SRH were 2.65 times higher among the respondents who had mobility impairment in reference to their counterparts [AOR: 2.65; CI: 2.42–2.89]. Older respondents having difficulty in ADL or IADL had 2.04 and 1.92 times higher odds of poor-SRH than their counterparts who did not have any difficulty in ADL or IADL, respectively.

**Table 3:** Logistic regression estimates of poor-SRH for older adults by their background characteristics in India, LASI (2017–2018)

	UOR	(95% CI)	AOR	(95% CI)
Background Characteristics	(Chi-square, p-value)		(Chi-square, p-value)	
**Migration Status**				
Non-migrant	Ref		Ref	
Migrant (10 years or more)	1.20 (< 0.001)	1.14, 1.27	1.08 (0.02)	1.01, 1.15
Migrant (less than 10 years)	1.43 (< 0.001)	1.25, 1.65	1.25 (< 0.001)	1.10, 1.50
**Mobility Impairment**				
No	Ref		Ref	
Yes	4.39 (< 0.001)	4.04, 4.78	2.65 (< 0.001)	2.42, 2.89
**ADL difficulty**				
No	Ref		Ref	
Yes	3.13 (< 0.001)	2.95, 3.32	2.04 (< 0.001)	1.91, 2.18
**IADL difficulty**	Ref			
No	2.72 (< 0.001)	2.58, 2.87	Ref	
Yes			1.92(< 0.001)	1.81, 2.04
**Age**				
Young-old (60–69)			Ref	
Old-old (70–79)			1.27 (< 0.001)	1.18, 1.35
Oldest-old (80+)			1.76 (< 0.001)	1.60, 1.94
**Sex**				
Male			Ref	
Female			0.82 (< 0.001)	0.77, 0.88
**Education**				
Higher			Ref	
No education/Primary			1.71 (< 0.001)	1.49, 1.94
Secondary			1.49 (< 0.001)	1.30, 1.70
**Marital Status**				
Married			Ref	
Widowed			1.11 (0.55)	0.78, 1.56
Others			1.82 (0.01)	1.24, 2.63
**Living arrangement**				
Living alone			Ref	
Living with spouse			0.78 (0.18)	0.78, 1.56
Living with others			0.71 (< 0.001)	0.62, 0.80
**Working Status**				
Working			Ref	
Not working			1.47 (< 0.001)	1.36, 1.60
**Social Participation**				
Yes			Ref	
No			1.11 (0.09)	0.98, 1.26
**Physical Activity**				
Rigorous			Ref	
Moderate			1.19 (0.01)	1.06, 1.35
Never			1.88 (< 0.001)	1.70, 2.07
**Yoga meditation**				
Daily			Ref	
Often			1.02 (0.81)	0.85, 1.22
never			1.48 (< 0.001)	1.34, 1.64
**Multi Morbidity**				
No			Ref	
Yes			2.56 (< 0.001)	2.40, 2.73
**Sleep Problem**				
No			Ref	
Yes			1.62 (< 0.001)	1.53, 1.71
Pain				
No			Ref	
Yes			2.16 (< 0.001)	2.04, 2.29
**MPCE Quintile**				
Highest			Ref	
Lowest			1.10 (0.04)	0.99, 1.21
Lower			1.03 (0.49)	0.92, 1.36
Middle			0.90 (0.03)	0.82, 0.99
Higher			1.06 (0.30)	0.96, 1.16
**Religion**				
Hindu			Ref	
Muslim			0.99 (0.88)	0.91, 1.09
Others			0.81 (< 0.001)	0.74, 0.89
**Caste**				
Others			Ref	
SC/ST			1.04 (0.39)	0.96, 1.12
Other backward class			1.28 (< 0.001)	1.19, 1.37
**Residence**				
Urban			Ref	
Rural			1.18 (< 0.001)	1.10, 1.26
**Pseudo R2**			0.1305	
**Chi-square, p-value**			< 0.001	

Ref: Reference, UOR: Unadjusted Odds ratio, AOR: Odds ratio adjusted for all the selected covariates; CI: Confidence Interval,SC/ST: Schedule caste/ Schedule tribe, OBC: Other backward class, MPCE: Monthly per capita consumption expenditure

Furthermore, increasing age was found to be positively associated with poor-SRH and was highest for the age group of 80 + years [AOR: 1.76; CI: 1.60–1.94] as compared to the age group of 60–69 years. The odds of reporting poor-SRH were higher among the respondents with no/primary education [AOR: 1.71; CI: 1.49–1.94] than their counterparts who had higher education. Older adults who were living with others were lower likely to report poor-SRH in comparison with the older adults who were living alone [AOR: 0.71 CI: 0.62–0.80]. Older adults who were currently not working [AOR: 1.47; CI: 1.36–1.60] and never did any physical activity [AOR: 1.88 CI: 1.70–2.07] were more likely to report poor-SRH than their counterparts who were currently working or did rigorous physical activity, respectively. The odds of reporting poor-SRH increased when the participants were suffering from multi morbidity than those who did not have multi morbidity [AOR: 2.56; CI: 2.40–2.73]. The odds of reporting poor-SRH were 1.62 times [AOR: 1.62; CI: 1.53–1.71] and 2.16 times [AOR: 2.16; CI: 2.04–2.29] higher in older adults who were suffering from sleep problem and chronic pain than their counterparts who were free from these problems. The odds of reporting poor-SRH were higher among rural respondents [AOR: 1.18; CI: 1.10–1.26] compared with their urban counterparts.

Table 4 presents the adjusted multitude effects of migration status and mobility impairment on the odds of reporting poor-SRH among older adults. Recent migrants living for less than ten years in the destination place with a mobility impairment had 3.39 times higher odds of poor-SRH than non-migrants without mobility impairment [AOR: 3.39; CI: 2.79–4.13]. Likewise, migrants with a longer duration (living in the place for ten or more years) with mobility limitation also had higher odds of poor-SRH than their non-migrant counterpart with no mobility limitation [AOR: 2.87; CI: 2.52–3.27].

**Table 4:** Stratification of migrant status and mobility impairment and its association with poor-SRH

Migration status # mobility impairment	AOR (p-value)	(95% CI)
Non migrant# no	Ref	
Non migrant# yes	2.78 (< 0.001)	2.45, 3.15
Migrant for 10 or more years# no	1.14 (0.12)	0.97, 1.35
Migrant for 10 or more years# yes	2.87 (< 0.001)	2.52, 3.27
Migrant for less than 10 years# no	1.36 (0.16)	0.88, 2.11
Migrant for less than 10years# yes	3.39 (< 0.001)	2.79, 4.13
**Pseudo R2**	0.1299	
**Chi-square, p-value**	< 0.001	

Ref: Reference, AOR: Odds ratios are adjusted for all the selected covariates,CI: Confidence Interval

Table 5 shows the estimates from the adjusted stratified analysis of migration status and difficulties in ADL. Both recent migrants (less than 10 years) [AOR: 2.44; CI: 1.87–3.20] and migrants with ten or more years of staying in the destination place [AOR: 2.12; CI: 1.93–2.33] having difficulty in ADL had higher odds of poor-SRH than their non-migrant counterparts who did not have any difficulty in ADL.

**Table 5:** Stratification of migrant status and difficulty in ADL and its association with poor-SRH

Migration status # Difficulty in ADL	AOR (p-value)	(95% CI)
Non migrant# no	Ref	
Non migrant# YES	2.10 (< 0.001)	1.89, 2.33
Migrant for 10 or more years# no	1.06 (0.15)	0.98, 1.14
Migrant for 10 or more years# yes	2.12 (< 0.001)	1.93, 2.33
Migrant for less than 10 years# no	1.28 (0.01)	1.07, 1.53
Migrant for less than 10years# yes	2.44 (< 0.001)	1.87, 3.20
**Pseudo R2**	0.1275	
**Chi-square, p-value**	< 0.001	

Ref: Reference, AOR: Odds ratios are adjusted for all the selected covariates,CI: Confidence Interval

A similar result came up (Table 6) when we stratified the migration status and difficulty in IADL activities, adjusting for other covariates. Migrants (less than ten years) who had difficulty in IADL had 2.22 times higher odds of poor-SRH compared to non-migrants without any difficulty in IADL [AOR: 2.22; CI: 1.79–2.74]. On the other hand, migrants with a longer duration of residence at destination place with difficulty in IADL also had higher odds of poor-SRH [AOR: 2.04; CI: 1.86–2.23] in comparison with non-migrants without any such difficulties.

**Table 6:** Stratification of migrant status and difficulty in IADL and its association with poor-SRH

Migration status # Difficulty in IADL	AOR (p-value)	(95% CI)
Non migrant# no	**Ref**	
Non migrant# yes	1.86 (< 0.001)	1.70, 2.04
Migrant for 10 or more years# no	1.02 (0.61)	0.93, 1.13
Migrant for 10 or more years# yes	2.04 (< 0.001)	1.86, 2.23
Migrant for less than 10 years# no	1.36 (0.01)	1.10, 1.69
Migrant for less than 10years# yes	2.22 (< 0.001)	1.79, 2.74
**Pseudo R2**	0.1278	
**Chi-square, p-value**	< 0.001	

Ref: Reference, AOR: Odds ratios are adjusted for all the selected covariates,CI: Confidence Interval

## Discussion

This study examined the self-rated health status of older Indian adults according to their migration status, functional limitations and mobility impairment. The findings of the study reflect current challenges for older adults that will arise due to large-scale migration as India’s demographic and socioeconomic transition continues [51]. The older population is not a homogeneous group; they have differences in their physical, physiological and socio-cultural situations. These older adults have strong attachments with their birthplaces, which raise concerns during geographical relocation [52]. As an individual’s identities are mostly linked to his/her place of birth [53], leaving one’s home is a potential cause of emotional distress. Findings from this study show that the proportion of migrants reporting poor health status was significantly higher (25.14% among those who are migrants for the last ten years or more and 28.03% among those who are migrants for less than ten years) than non-migrants (22.75%). Several previous studies also noted a greater proportion of migrants reporting poor health status among older migrants as compared to non-migrants [18, 20]. On the contrary, some studies have found no clear trend of deteriorating health status of migrant older adults [18, 19].

Furthermore, mobility impairments become more common as people get older, and they are linked to poor health outcomes. Numerous studies among older adults showed that the overall prevalence of mobility impairment ranged within 22.5–46.7%. [54–57]. Our findings showed that the prevalence of poor-SRH among those with mobility limitations was 28.65%, which is similar to other studies where mobility issues have been linked to an increased risk of falling, poor health status, and a lower quality of life [58–62]. Moreover, older adults with mobility impairment were more likely to report poor SRH than those without mobility impairment. Multiple previous studies shows strong links between poor daily activities, risk of fall, hospitalisation, mental health issues, quality of life, and even mortality among older people with mobility issues [58, 59, 63, 64].The ability to perform ADLs and IADLs are an important measure of an individual’s functional status, as it reflects their capacity to complete basic daily tasks. Our finding reported greater proportion of respondents with limited ADL reported poor-SRH than those without the limitations. This finding is supported by studies from different countries showing individuals who reported difficulties performing ADLs are more likely to report poor-SRH [65–70]. ADL limitations can impact an older adult’s overall quality of life and sense of well-being. The individual experiencing problems with ADL bears loss of independence, and a reduced ability to participate in meaningful activities. This results in detrimental impact on the self-perception of their health status, contributing to the likelihood of reporting poor- SRH. Similarly, our study found that a higher percentage of those with more complex daily activities i.e. IADL difficulties reported poor-SRH than those without the constraints. Previous studies also found IADL limitations to be significant risk factors for poor-SRH [71, 72]. Yet, there may be bidirectional relationships between limitations in ADL/IADL and poor-SRH; as limitations in ADL/IADL can be a result of poor-SRH and vice versa [72].

Mobility impairment was an important factor among older adults that amplify the poor SRH condition. Results of the study revealed that those who were having mobility impairment were more than twice as likely to experience poor health conditions which is consistent with findings from other research [62–64]. Further, the study exhibits that in the combined effect of migrants with mobility impairment, accentuated the effect of poor SRH around four folds as compared to non-migrants older adults without any mobility impairment. Poor SRH was reported by 54.42% of migrant older adults with mobility impairment. Prior studies demonstrated that poor health status was associated with migration and mobility impairment [64, 73–77]. It is noteworthy that in terms of health, studies shows that migrants have poorer health than non-migrants, despite the “healthy migrant effect,“ which indicates that the average health of immigrants is greater at least during the time of movement [78–81]. However, migration can also present challenges for older adults with ADL and IADL problems. This study investigated the association between migration status and ADL problems and estimated that both recent migrants (less than ten years) and migrants with ten or more years of staying in the destination place with ADL problems were more than twice as likely to report poor-SRH as their non-migrant counterpart with no ADL problems. As adjusting to a new location can be stressful, especially if the older adult is leaving behind familiar surroundings and support networks [82]. Additionally, older adults who require assistance with ADLs may face additional barriers to migration, such as limited mobility or financial constraints [83]. Consequently, when ADL/IADL problems exist among older adults with a migration background, they are most likely to use the informal care. As person with a migratory past may lack experience in the care system, and have cultural and linguistic limitations [84].

Interestingly, the findings on the relationship between migration status and IADL limitations indicated that both recent migrants (less than ten years) and migrants with ten or more years of staying in the destination place with IADL problems were more than twice as likely as their non-migrant counterparts with no IADL problems to report poor-SRH similar to other studies [85, 86]. However another study found no evidence of significant moderating effect of migration status on the link between ADL/IADL limitations and the care utilisation [84]. Also migration can offer older adults the opportunity to receive better care and support from family members or social service programs. For example, migrating to a location with better public transportation systems can provide older adults with greater independence and mobility. Additionally, older adults who migrate to a location with a higher quality of community services, such as meal delivery or home repair programs, may experience improved quality of life [87, 88].

In general, SES indicators provide information regarding access to social and economic resources. According to our findings, poor/disadvantageous socioeconomic and demographic factors increased the likelihood of poor SRH in migrant older adults compared to non-migrants, which is supported by a number of studies with similar findings [19, 89, 90]. A narrative review of the health profile of the aging migrants shows health risks before and during migration. Migrants’ disadvantageous socioeconomic status, cultural factors influencing health-seeking behaviours, and psychosocial vulnerability and discrimination all have an impact on their health and quality of life [89]. Other research suggests that older migrants view their health conditions as being poorer than the non-migrant people, despite the fact that a country of origin is an important factor determining differences in health conditions among migrants and how they utilise health services [87]. Moreover, adequate healthcare approaches are frequently based on the sociological and legal nature of migrants, making it one of the most important drivers [87, 91]. However, on contrary, a study shows older adults seek migration to countries to access better health care facilities and better quality of life [87].

Age of older adults being an important indicator, is examined in this population-based study, shows that age was found to be positively associated with poor SRH and was highest in the 80 + age group as compared to older adults in the 60–69 age group. A research conducted by Kaluza–Kopias on people aged 75 years or older, shows that around 88% of aged people did not want to migrate or relocate because of the concern for poor health conditions [92]. Further, respondents with no/primary education were more likely to report poor SRH than those having higher education. A closer look at the literature reveals that education has a significant impact on the health of older people [19, 93, 94].

In addition, our results showed that not-working older adults were more likely to report poor SRH. Working status is an indicator of financial condition and study shows older migrants are positively associated with their relative income status and they are more likely found to have a low income [95]. Yet, interestingly, a study found that Hispanics who immigrated at the age of 18 still had sharp declines in health condition after the age of 50 years, because of discrimination, underinsurance and low-paying work environment [20]. Furthermore, migrant older adults have lower physical activity, which leads to poor SRH [96]. Moreover, another interesting aspect is that better midlife working ability of a person may protect SRH among those who retire due to old age mobility limitations [97]. So, promoting work ability in middle age may result in better SRH. Additionally, our study also found older adults who practiced yoga meditation on daily basis were less likely to report poor SRH than those who never practiced yoga. Several studies have backed these claims, that meditation and yoga can help reduce stress, PTSD, healthy sleep pattern, better mood, good memory, and improve overall health. Concordantly, recent interventional studies revealed that older people over the age of 60 who participated in a yoga interventional groups notably improved their stress biomarkers as well as their physical mobility [98, 99].

Our findings are consistent with previous studies where multiple morbidities have a higher likelihood of reporting poor health compared to native-born populations [100, 101]. Migration necessitates acclimating to new healthcare systems, where multi morbidity and susceptibility to infection may be a concern, and mobility impairment further exacerbates the situation [87]. However refuting previous research, two nationwide studies from Norway and Sweden confirm that migrants have lower multi-morbidity levels as compared to non-migrants [102, 103].Further, our findings of sleep problems and chronic pain among the migrant ageing population suggest to have reported poor SRH and significant relationship have been consistent and well documented in numerous previous studies [104–108].

Migration among older population can be a response to poverty, and as part of family diversification, many times it can lead to social exclusion experiencing a stressful impact. Further many studies including ours establish older adults from other backward castes when migrate were more likely to report poor SRH [109]. In addition, our findings show older adults from rural areas augmented to report inadequate SRH similar to results encountered in other parts of the world [87, 110]. Other research suggests multiple factors influence the SRH, including neighbourhood, residence, health services, amenities, mobility status, social relations, material and financial resources, socio-cultural aspects, and civic participation [111]. Migration along with mobility issues have a lot of potential for explaining and responding to disadvantages in life later.

There are few limitations to the current study. First, because findings of this study are based on cross-sectional data, we could not establish causality between migration and mobility limitation with SRH. Second, self-reported information about chronic conditions and mobility limitations may be inaccurate, resulting in information bias. However, the study has its own strengths, as our findings, for the first time provide evidence on the effect of migration and the contribution of mobility limitation on SRH at the national level. In addition, the study has a large sample size and the data is rich in information on older adults’ self -reported health status and ageing-related issues in the Indian context.

## Conclusions

The findings revealed that older adults who have ever migrated and have functional or mobility impairment have much higher odds of perceiving their general health as poor in comparison to non-migrant counterparts with no mobility impairment. The findings also suggest the vulnerability of migrant older adults with functional or mobility disability, as well as those with limited socioeconomic resources and suffering from multimorbidity. The findings can be utilised to target outreach programmes and provision of services for migrating older individuals with mobility impairments.

## Data Availability

The study uses a secondary data which is available on reasonable request through https://www.iipsindia.ac.in/content/lasi-wave-i.
